# Prevalence of pulmonary tuberculosis in young adult patients with Type 1 diabetes mellitus in India

**DOI:** 10.1186/s40248-016-0058-z

**Published:** 2016-05-10

**Authors:** Abilash Nair, Randeep Guleria, Devasenathipathy Kandasamy, Raju Sharma, Nikhil Tandon, Urvashi B. Singh, Ravinder Goswami

**Affiliations:** Department of Endocrinology and Metabolism, All India Institute of Medical Sciences, New Delhi, 110029 India; Department of Microbiology, All India Institute of Medical Sciences, New Delhi, 110029 India; Department of Pulmonary Medicine, All India Institute of Medical Sciences, New Delhi, 110029 India; Departments of Radiodiagnosis, All India Institute of Medical Sciences, New Delhi, 110029 India

**Keywords:** Type1 Diabetes, Tuberculosis, HbA1c

## Abstract

**Background:**

There is limited information on prevalence of pulmonary tuberculosis (PTB) in patients with type-1-diabetes. We assessed the prevalence of PTB in patients with type-1-diabetes attending the outpatient-clinic in a tertiary-care hospital.

**Methods:**

151 patients with type-1-diabetes were screened for PTB by clinical examination and chest-radiography. Sputum Acid-Fast Bacilli Test (AFB) and *Mycobacterium tuberculosis* (*M.tb*) culture were performed in patients with clinical and radiological features suggestive of a possibility of PTB and also in those with history of PTB in the past. Their average glycated haemoglobin (HbA1c) during preceding 2 years was assessed. Sputum culture positive patients were managed by a pulmonologist.

**Results:**

5/151 patients had respiratory symptoms and radiographic findings suggestive of PTB. 20/151 patients were asymptomatic but had history of PTB. Four of the five symptomatic patients and 12 with past PTB were positive for sputum *M.tb* by culture, giving a prevalence of 10.6 % sputum culture positive in type-1-diabetes. Average HbA1c was comparable in patients with and without positive sputum culture. ESR and Mantoux test were not discriminatory in these groups. Four clinically symptomatic *M.tb* culture positive and four asymptomatic patients with sputum culture positive for *M.tb* on two occasions (6 weeks apart) were put on antitubercular treatment (ATT). Patients who were culture positive for *M.tb* only on one occasion were kept on a close follow up.

**Conclusions:**

Patients with type-1-diabetes mellitus in India have high prevalence of PTB. They need to be actively screened for PTB by sputum *M.tb* culture in order to initiate early treatment and to prevent transmission in the community.

## Background

Association between diabetes mellitus and tuberculosis is known since the mid-twentieth century and has been increasingly drawing attention [1-3]. There is 3.5–5.0 fold higher risk of tuberculosis in diabetes, which is especially high in type 1 diabetes [4, 5]. These patients have high relapse after antitubercular drugs and high mortality in case of delayed diagnosis. The World Health Organization recommends that patients with diabetes and cough of > 2 weeks need further evaluation for possibility of tuberculosis [6].

Recently, Lin et al. screened 3,087 patients with type 2 diabetes and detected 11 fresh cases of pulmonary tuberculosis; indicating relevance of WHO recommendations [7]. There is paucity of similar information in type 1 diabetes mellitus. Tuberculosis is common in India with prevalence of 256/100,000 persons. In such a scenario, likelihood of tuberculosis might be high in patients with diabetes mellitus. The present study assessed the prevalence of pulmonary tuberculosis (PTB) in a cohort of patients with type 1 diabetes attending a tertiary care hospital in North India and its correlation with glycemic status maintained during the preceding 2 years.

## Methods

Study subjects were insulin-requiring patients with age of onset of diabetes < 30 years, attending the ‘Diabetes of young’ clinic at All India Institute of Medical Sciences, New Delhi. The clinic is operating since 1989–90 on weekly basis and has a weekly attendance of 30–40 patients. The study was carried out during December 2014-June 2015. All the new and follow up patients attending the clinic between 8.30 and 9.30 am were included so as to complete the study investigations on the same day. Pregnant and lactating female patients were excluded. Clinical details including age of onset of diabetes, presence of albuminuria, retinopathy and HbA1c values available during the preceding 2 years were recorded from clinic records. All the patients were individually assessed for symptoms and signs suggestive of active PTB such as fever, cough > 2 weeks, hemoptysis and noticeable weight loss during past six months. Past history of treatment for tuberculosis was also recorded. All the patients were recruited after written informed consent. The study protocol was approved by the Institutional review board of All India Institute of Medical Sciences, New Delhi. All procedures followed were in accordance with the ethical standards of the responsible committee on human experimentation (institutional and national) and with the Helsinki Declaration of 1975, as revised in 2008.

Digital chest radiograph, complete blood count, erythrocyte sedimentation rate (ESR), HbA1c and tuberculin skin test (TST) was performed for all the patients on their first visit. TST was performed with 2 tuberculin units of purified protein derivative (Span diagnostics, Surat, India) given intradermally on the volar aspect of the left forearm. The transverse diameter of induration was measured after 48–72 h using a ball-point pen and a transparent ruler. Patients were considered to have latent tuberculosis infection when induration was ≥10 mm with no other features suggestive of active tuberculosis.

The definitions of the PTB were made as per the definition and reporting framework of WHO criteria [6]. Briefly, patients with clinical symptoms suggestive of active tuberculosis, radiographic findings such as consolidation, fibrocavitary disease and hilar lymphadenopathy and those with history of treatment for tuberculosis in the past were advised to bring a morning sputum specimen for AFB smear, *M.tb* culture and antitubercular drug susceptibility testing (DST). A second sputum sample was collected in the clinic. Patients who were unable to expectorate were given nebulization with 3 % saline solution for 10 min in an isolated area to induce sputum. Clinically asymptomatic patients with history of treatment for tuberculosis in the past or features of healed tuberculosis on chest radiography with sputum *M.tb* culture positive in any of the two sputum samples were further followed by a pulmonologist. For these patients, two more sputum cultures for *M.tb* and chest radiography were performed after six weeks. The World Health Organization approved rapid diagnostics, such as Xpert MTB/RIF, was not used for diagnosis in this study cohort for logistic reasons [6].

Sputum AFB smear was stained by Ziehl-Neelsen technique. *M.tb* culture and DST for rifampicin and isoniazid were performed on Middlebrook 7H10 agar [8]. Complete blood count, ESR and chest-radiography were performed in the general hospital departments of the hospital. Glutamic acid decarboxylase autoantibodies (Normal < 1.0 units/ml) were assayed in the hormone service laboratory of the endocrine Department.

### Follow up and treatment for patients with sputum *M.tb* culture positive

#### Symptomatic patients

Patients with clinical symptoms and/or chest radiograph suggestive of active pulmonary lesion with sputum smear or culture positive for *M.tb* were defined as clinically active PTB cases and were initiated on ATT. Those with symptoms of chest infection but without sputum smear or culture positive were given a course of antibiotics therapy other than fluoroquinolones. If the symptoms persisted after two weeks and repeat sputum smear or culture yielded a positive result, were also considered as bacteriologically active PTB cases and were initiated on ATT.

#### Clinically asymptomatic patients

Clinically asymptomatic patients who had *M.tb* culture positive in at least two sputum samples taken six weeks apart were advised ATT. Patients who developed symptoms or radiographic findings suggestive of tuberculosis during follow up were also put on ATT irrespective of repeat sputum *M.tb* culture. Patients with no clinical symptoms and normal chest radiograph who had only one sputum culture positive for *M.tb* were designated as subclinical PTB cases and advised regular follow up in the pulmonology clinic.

### Statistical analysis

Data are shown as mean ± SD and frequencies in percentage. The clinical and biochemical characteristics of patients with and without PTB were compared by parametric and nonparametric tests as appropriate. All statistical analyses were imputed on SPSS 16.0 (SPSS Inc., Chicago, IL, USA). A two tailed *p* of < 0.05 was considered significant.

## Results

**Fig. 1:**
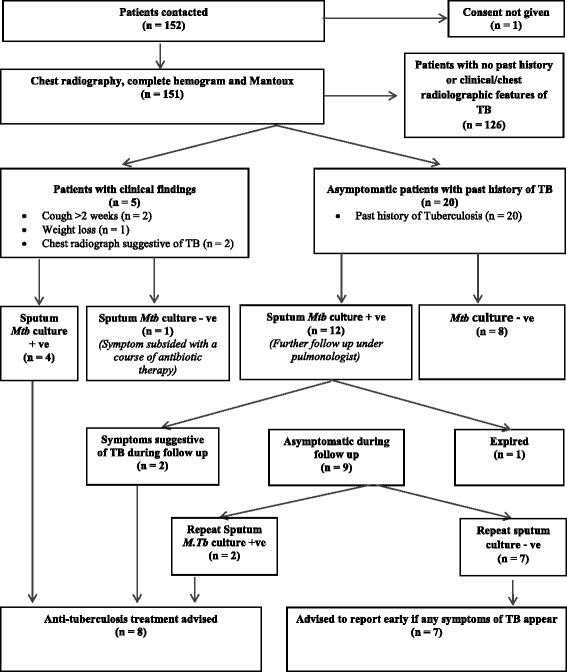
Flow of patients in the study

Figure 1 shows the flow of 152 patients in the study. One of them did not agree to participate and therefore, was excluded. Final analysis was carried out in 151 patients (males 76, females 75). History of spontaneous diabetic ketoacidosis during the course of the disease was present in 105 subjects. GAD_65_ autoantibodies were tested in 136 subjects and were positive in 47.1 %. Their mean age, duration of diabetes from onset of symptoms and BMI were 28.2 ± 11.22 years, 13.2 ± 7.54 years and 20.8 ± 3.74 kg/m^2^ respectively. History of smoking and alcohol intake was present in one each. Their mean HbA1c at screening and average of the last four values available during follow up in diabetic clinic were 9.0 (75 mmol/mol) ± 1.8 % and 9.0 (75 mmol/mol) ± 2.1 %, respectively. Retinopathy, urinary total protein excretion >150 mg/24 h and chronic kidney disease (estimated glomerular filtration rate < 60 ml/min) were present in 22 %, 8 % and 8.6 % respectively. The mean hemoglobin and total leukocyte count were 12.7 ± 2 gm% and 7435 ± 1877/mm^3^, respectively. The mean erythrocyte sedimentation rate of the study group was 18.1 ± 15.77 mm/hour and a total of 60 patients had elevated ESR (≥20 and ≥ 15 mm/hr for females and males respectively).

### Symptoms, chest radiograph and sputum *M.tb* culture at presentation

Clinical features suggestive of active PTB were present in five of the 151 patients screened, though they did not see the physician for these symptoms. These five included two with cough for more than two weeks (one with fibrocavitary consolidation on chest radiography and the other with normal radiography); one with significant weight loss and left upper zone cavity on chest radiography; two without symptoms but chest radiography suggestive of tuberculosis (one with consolidation and the other with hilar adenopathy). Twenty patients had no overt clinical features of PTB but had history of tuberculosis in the past. The details of all these 25 patients including their ESR, Mantoux, chest radiography and status of sputum *M.tb* culture on initial screening is given in Table 1.

**Table 1:** Clinical findings of 25 patients screened for sputum *M.tb* positivity and their follow up in the pulmonology clinic

S N	Age/Sex	Findings at initial screening						Follow up in the Pulmonology clinic				
		Indication for screening by sputum *M.Tb* culture		Sputum culture *M.tb*		ESR mm/hr	TST mm	Symptom	Radiograph	Sputum culture M.Tb		ATT
		History	Radiography	Ist	2nd					Ist	2nd	
1	40,M	Wt loss, Past TB	Lt upper lobe Cavity	+ve	+ve	40	11	None	No change	-ve	+ve	Yes
2	25,F	Cough > 2 weeks & Past TB	Lt upper lobe fibrocavitory consolidation	+ve	+ve	30	11	Hemoptysis	No change	-ve	+ve	Yes
3	36,F	Past TB	Lt upper lobe Consolidation	+ve	+ve	37	11	Wt loss	No change	-ve	-ve	Yes
4	28,F	None	Rt hilar adenopathy	+ve	-ve	8	5	None	No change	-ve	+ve	Yes
5	25,F	Past TB	Normal	+ve	-ve	38	10	Fever > 2 weeks	Normal	-ve	+ve	Yes
6	22,F	Past TB	Normal	-ve	+ve	118	5	Pericardial effusion	Pericardial effusion	ND	ND	Yes
7	28,F	Past TB	Rt pleural thickening	+ve	+ve	15	9	None	No change	-ve	+ve	Yes
8	47,F	Past TB	Rt bronchiectasis	+ve	SNA	26	10	None	No change	-ve	+ve	Yes
9	65,F	Past TB	Lt upper lobe Fibrosis	+ve	-ve	20	8	None	No change	-ve	-ve	No
10	39,M	Past TB	Normal	SNA	-ve	10	8	None	Expired			No
11	37,F	Past TB	Normal	+ve	SNA	5	0	None	Normal	-ve	-ve	No
12	40,F	Past TB	Rt lower lobe fibrosis	-ve	+ve	5	6	None	Normal	-ve	-ve	No
13	46, M	Past TB	Normal	+ve	-ve	30	4	None	Normal	-ve	-ve	No
14	36,M	Past TB	Normal	+ve	SNA	15	0	None	Normal	-ve	-ve	No
15	32,M	Past TB	Normal	+ve	+ve	10	2	None	Normal	-ve	-ve	No
16	40,M	Past TB	Normal	-ve	+ve	10	0	None	Normal	-ve	-ve	No
17	22,M	Cough > 2 weeks	Normal	-ve	-ve	3	6	None	NR	NR	NR	No
18	43,M	Past TB	Rt upper lobe fibrosis & Lt lower lobe calcification	-ve	-ve	5	10	None	NR	NR	NR	No
19	34,F	Past TB	Normal	-ve	-ve	16	5	None	NR	NR	NR	No
20	23,F	Past TB	Normal	-ve	-ve	10	4	None	NR	NR	NR	No
21	38, M	Past TB	Normal	-ve	-ve	8	0	None	NR	NR	NR	No
22	20,M	Past TB	Normal	-ve	-ve	10	10	None	NR	NR	NR	No
23	38,M	Past TB	Normal	-ve	-ve	5	12	None	NR	NR	NR	No
24	30,F	Past TB	Normal	-ve	-ve	48	0	None	NR	NR	NR	No
25	28,F	Past TB	Rt pleural thickening	-ve	-ve	3	0	None	NR	NR	NR	No

*M* male, *F* female, *Lt* left, *Rt* right, *TB* tuberculosis, *wt* weight, *SNA* sample not available, *ND* not done, *NR* not required

None of them had sputum smear positive for AFB. However, 16 of them had sputum *M.tb* culture positive in at least one of the two sputum specimens. All *M.tb* cultures were sensitive to rifampicin and isoniazid. ESR was elevated in significantly higher proportion of patients with positive *M.tb* culture as compared to those with negative culture (10/16 *vs.* 1/9, *P* = 0.03). TST was not significantly different in these two groups (5/16 *vs*. 3/9, *p* = 0.99). Four out of the five patients with clinical symptoms or chest radiography suggestive of PTB had positive *M.tb* culture. The fifth patient had cough, which responded to a course of oral antibiotics. Among twenty asymptomatic patients who had past history of tuberculosis, twelve had positive sputum *M.tb*. culture.

### Clinical correlates of sputum *M.tb* culture positive patients

Table 2 shows the clinical characteristics of 16 patients with sputum *M.tb* culture positive in comparison to those in whom clinical, radiological and bacteriological features were not suggestive of PTB (*n* = 135). Patients with positive *M.tb* culture were older on an average by 10 years (36.9 ± 10.5 *vs.* 27.1 ± 10.87 years, *p* < 0.001) and had significantly higher duration of diabetes (18.9 ± 5.38 vs. 12.5 ± 7.46 years, *p* < 0.01). Retinopathy and chronic kidney disease were present in higher proportion of patients with positive culture (*p* < 0.001 for both). The mean HbA1c at the time of screening was comparable in two groups (8.1 (65 mol/mol) ± 1.7 *vs.* 9.1 (76 mmol/mol) ± 2.1 %, *p* = 0.10). The total numbers of HbA1c values available during past 2 years were 54 for the 16 patients with positive culture and 430 in others. The mean HbA1c of all pooled values were also not significantly different in these two groups (8.4 (68 mmol/mol) ± 1.7 % *vs.* 9.1 (76 mmol/mol ± 1.8 %, *p* = 0.19). Other clinical and biochemical parameters were comparable in both groups (Table 2).

**Table 2:** Baseline characteristics in patients with and without positive sputum *M.tb culture*

Parameters	Sputum *M.Tb* status		*P*
	Negative	Positive	
	(*n* = 135)	(*n* = 16)	
Male: Female (n)	65:70	10:6	0.59
Age (years)	27.1 ± 10.87	36.9 ± 10.5	<0.001
Body mass index (kg/m^2^)	20.8 ± 3.78	20.9 ± 3.36	0.89
Duration of diabetes (years)	12.5 ± 7.46	18.9 ± 5.38	<0.01
Retinopathy (n)	22 (16.29 %)	11(68.75 %)	<0.001
Proteinuria(urine protein >150 mg/24 h)	18 (13.33 %)	4 (25.0 %)	0.22
Chronic kidney disease (eGFR <60 ml/min)(n)	8 (5.5 %)	5 (31.2 %)	<0.001
Current HbA1c (%)	9.1 ± 2.1	8.1 ± 1.7	0.10
Pooled HbA1c during (%)	9.1 ± 1.8	8.4 ± 1.7	0.19
Serum total cholesterol (mg/dL)	165.8 ± 36.68	171.8 ± 32.75	0.53
Serum triglycerides (mg/dL)	98.3 ± 54.58	107.7 ± 47.42	0.51
Serum LDL (mg/dL)	93.2 ± 28.98	97.9 ± 27.32	0.53
Serum HDL (mg/dL)	54.1 ± 19.28	51.3 ± 14.47	0.58
Hemoglobin (g/dl)	12.7 ± 2.04	12.8 ± 1.65	0.90
Total leukocytes count/mm^3^)	7360 ± 1889	8069 ± 1695	0.15
Polymorphs (%)	59.4 ± 9.75	61.1 ± 9.66	0.52
Lymphocyte (%)	30 ± 8.1	28 ± 9.1	0.20
Erythrocyte sedimentation rate (mm/h)	17.0 ± 13.62	27.1 ± 26.80	<0.001
Elevated ESR (*n* = 147)	50 (38.2 %	10 (62.5 %	0.06
Mantoux (>10 mm) (*n* = 145)	13 (10.07 %)	5 (31.25 %)	0.02

### Follow up and treatment of patients for PTB

Table 1 gives the follow up details of all the 16 patients who had positive sputum *M.tb* culture. Four of them with symptoms or chest radiography suggestive of tuberculosis at presentation were advised ATT. Other patients were kept under close clinical monitoring for the occurrence of symptoms suggestive of tuberculosis. Repeat sputum culture was carried out after six week interval. Two patients including one who developed pericardial effusion and another who developed prolonged fever within six weeks were advised ATT. One patient who also had advanced nephropathy died at his native village home before completion of six week follow up. Two patients though asymptomatic, again had positive sputum *M.tb* culture at six weeks and were put on ATT. Seven patients who remained asymptomatic and did not grow *M.tb* on repeat sputum cultures were advised to remain under regular follow up in the pulmonology clinic.

## Discussion

In the present study, we actively screened for the presence of PTB among patients with type 1 diabetes, who were on routine follow up in the endocrine clinic at a tertiary care hospital. Besides, all the patients with positive *M.tb* sputum culture were prospectively followed up by an expert pulmonologist for assessing the need for instituting ATT. Despite its clinical relevance, there is paucity of such information with only one similar study available [9].

The present study revealed a high prevalence (10.6 %) of sputum *M.tb* culture positivity in type 1 diabetics. Most of the patients were clinically asymptomatic (87 %) and only two had symptoms suggestive of tuberculosis on direct questioning. In the present study, 60 % of the patients who had previous history of ATT, were currently asymptomatic but showed positive *M.tb* sputum culture. This indicated a high rate of persistence or relapse of disease in type 1 diabetes with past history of tuberculosis. The asymptomatic nature of the disease in most of the *M.tb* culture positive patients could be related to their low bacterial load as indicated by smear negativity on microscopy. Notwithstanding the low sputum *M.tb* load, 50 % of the patients with positive *M.tb* sputum culture required ATT in view of their symptoms or persistent culture positivity on follow up. Other patients with sputum *M.tb* culture positive but with no current symptoms were advised close follow up in the respiratory clinic as cases with ‘subclinical PTB’. ESR and Mantoux test could not reliably discriminate patients with and without *M.tb* culture positive. Interestingly, in the present study, sputum AFB was negative in all the patients including those in whom sputum culture was positive for *M.tb*. Tostmann et al., [10] showed that patients with smear-negative, culture-positive TB are responsible for 13 % of TB transmission in Netherland. Recently, Assael et al. [11] reported that 80 % Mexican immigrants to USA had smear negative TB, thereby suggesting a need to include sputum culture as the screening method for TB diagnosis. The results of the current study suggest the advantage of sputum *M.tb* culture over sputum AFB smear as screening test for diagnosis of tuberculosis in patients with type 1 diabetes as well.

In the present study, eight clinically asymptomatic patients with positive sputum *M.tb* culture, only on one occasion were put on follow up as subclinical PTB. In the absence of clear guidelines regarding management, pulmonologist deferred ATT therapy. However, two such patients presented with complications within 6 weeks, one developed pericardial effusion and another developed prolonged fever and were advised ATT. These patients were asymptomatic and hence were put on follow up. If ATT had been initiated, based on the first culture report, the disease progress could have been halted. Thus there is a need to carry out further studies and develop clear management strategy among asymptomatic patients with type 1 diabetes showing lower micobiological load of *M.tb*.

Patients with diabetes mellitus are at increased risk for active tuberculosis. Jeon and Murray performed a meta-analysis of 13 observational studies and reported that diabetes mellitus was associated with an increased risk of TB with relative risk of 3.11 (95 % CI 2.27–4.26) [12]. Such increased risk was postulated to be due to multiple abnormalities in the innate and adaptive immune system among diabetic subjects [13]. The higher prevalence of positive *M.tb* culture in our study population could also be due to other reasons. These include a higher background prevalence of tuberculosis among Asian Indians possibly related to socioeconomic factors such as overcrowding and malnutrition. Besides, the lower threshold for screening by including asymptomatic patients with past history of tuberculosis and use of sputum *M.tb* culture could be other reason for the higher prevalence of tuberculosis detected in the current study population.

Interestingly, despite higher duration of diabetes and micro-vascular complications in patients with culture positive than in those without, current or average of the HbA1c values during past 2 years were not significantly different. Webb et al. observed 3.5 % prevalence of symptomatic PTB in 258 patients with type 1 diabetes in South Africa, which correlated with duration of diabetes and poor glycemic control [9]. Leung et al. assessed 4690 elderly diabetic patients and observed a three times increased hazard of active tuberculosis in subjects with HbA1c greater than 7 % compared with those with HbA1c less than 7 % [14]. Though the above studies suggest that poor glycemic control seems to be a risk factor for tuberculosis, there is paucity of similar studies assessing relationship between glycemic status and prevalence of tuberculosis or sputum *M.tb* positivity. We had assessed mean HbA1c of the patients during the preceding 2 years. However, it would not accurately factor the extent of their dysglycemnia during their entire course of diabetes during which *M.tb* infection might have occurred. Moreover, a host of other abnormalities such as altered innate response reflected by reduced CD14^+^ subtype of monocytes and toll-like receptors-4 expression in the peripheral leukocytes and cell mediated immunity in diabetes might also affect the strength of association between HbA1c and presence of sputum *M.tb* [13, 15, 16]. In our earlier studies, we observed variable association between HbA1c and genitourinary infections in patients with diabetes mellitus [17, 18]. There is paucity of studies assessing relationship of glycemic control with tuberculosis or sputum *M.tb* positivity and this could be a subject for future studies.

Thus, to conclude, there is high prevalence of clinically overt and subclinical PTB in patients with type 1 diabetes. Physicians managing these patients need to actively screen them regularly with sputum culture in order to initiate early treatment and also to protect the community from exposure to TB transmission.

## Conclusions

There is limited information on prevalence of pulmonary tuberculosis (PTB) in patients with type-1-diabetes. We assessed the prevalence of PTB in 151 patients with type-1-diabetes attending the outpatient-clinic in a tertiary-care hospital. 10.6 % of them showed sputum *M.tb* culture positivity; especially in those with past history of PTB. Thus there is a high prevalence of PTB in patients with type1 diabetes in India. They need to be actively screened for PTB by sputum *M.tb* culture in order to initiate early treatment and to prevent transmission in the community.

## Statement of human and animal rights

All procedures followed were in accordance with the ethical standards of the responsible committee on human experimentation (institutional and national) and with the Helsinki Declaration of 1975, as revised in 2008.

## Statement of informed consent

Informed consent was obtained from all patients for being included in the study.
